# Targeting IL-12 for pancreatic cancer immunotherapy: advances in delivery strategies and clinical translation

**DOI:** 10.3389/fimmu.2026.1755796

**Published:** 2026-03-25

**Authors:** Jessica Schlatter, Wei Wei, YingYing Chen, Annick Melse, Yong Wang, Sara Guerra, Jose Oberholzer

**Affiliations:** 1 Department of Visceral and Transplant Surgery, University Hospital Zürich, Zürich, Switzerland; 2 Department of Information Technology and Electrical Engineering, ETH Zürich, Zürich, Switzerland

**Keywords:** cancer immunotherapy, interleukin-12, pancreatic ductal adenocarcinoma, tumour microenvironment, localized cytokine delivery, nanoparticle drug delivery, immune checkpoint inhibitors, oncolytic virotherapy

## Abstract

Pancreatic ductal adenocarcinoma (PDAC) remains highly resistant to conventional immunotherapies due to its immunosuppressive tumor microenvironment. Interleukin-12 (IL-12), a potent immunostimulatory cytokine, has demonstrated remarkable therapeutic potential across a range of malignancies. However, systemic administration of IL-12 has been linked to severe dose-limiting toxicities, including cytokine release syndrome and multi-organ dysfunction, representing a persistent barrier to clinical translation. This review examines the rationale for IL-12-based immunotherapy in PDAC, synthesizing the current knowledge on immunotherapy approaches for this disease, IL-12’s mechanisms of action in cancer treatment, and synergistic combination strategies. We describe three major delivery platforms that have emerged to overcome the limitations of systemic delivery due to toxicity: cellular encapsulation systems, virotherapy-mediated approaches, lipid nanocapsule formulations, polymer-based platforms, local electroporation-mediated transfection, and CAR-T combined therapy. These innovative strategies enable localized, sustained IL-12 expression within the tumor microenvironment while minimizing systemic exposure and associated toxicities. By safely harnessing IL-12’s potent immunostimulatory properties, these next-generation delivery systems offer promising therapeutic avenues for PDAC and other immunotherapy-resistant malignancies.

## 1 Introduction

Pancreatic cancer ranks as the 12th most common cancer globally, yet it is the 7th leading cause of cancer-related mortality (1). Alarmingly, projections suggest that by 2030, it will become the second leading cause of cancer mortality in the USA, demonstrating the urgent need for more effective treatments (2). Pancreatic ductal adenocarcinoma (PDAC) is the most prevalent form of pancreatic cancer, accounting for approximately 90% of cases (3). It arises from the epithelial cells lining the pancreatic ducts, most often in the head of the pancreas (4). PDAC is among the most aggressive carcinomas, characterized by rapid local invasion, early metastatic dissemination, and resistance to conventional therapies (5).

The major clinical challenge in the early detection of PDAC is the nonspecific symptoms in its early stages and the absence of reliable serological biomarkers (5). Carbohydrate antigen 19-9 (CA19-9) is currently the only U.S. Food and Drug Administration (FDA)- approved test for monitoring and management of pancreatic cancer (6). However, its sensitivity and specificity for detecting early-stage PDAC remain low (7–9). Currently, surgical resection remains the only potential curative option for PDAC (10). However, approximately 80% of patients are diagnosed with advanced-stage disease (stage III or IV). By this time, the disease has typically metastasized to distant sites, and curative surgery is no longer feasible (10).

Collectively, the aforementioned challenges mean that only 15-20% of patients are eligible for surgical resection at diagnosis (11–13). The majority must rely on alternative treatments, such as chemotherapy, radiotherapy, targeted therapy, or immunotherapy; however, PDAC presents significant therapeutic challenges due to widespread treatment resistance across these multiple modalities. Chemotherapy and radiotherapy face similar obstacles, including the dense desmoplastic extracellular matrix that acts as a physical barrier (14, 15). PDAC cells also engage intrinsic survival mechanisms that limit radiation-induced cell death (16, 17). Immunotherapy has also shown limited success against PDAC, as the immunosuppressive tumor microenvironment, characterized by dense stroma, immune exclusion, and immunomodulatory cells, prevents effective T-cell engagement (18–23).

The failure of diverse therapies in PDAC indicates that, despite systemic delivery, intratumoral efficacy was low due to the PDAC microenvironment. Although combination approaches and novel agents targeting the tumor microenvironment (TME) show promise, clinical translation remains challenging, highlighting the need for innovative therapeutic strategies that address the multifaceted nature of PDAC resistance and enhance antitumor immune responses. In this context, cytokine-based therapies, particularly interleukin-based therapies, have garnered significant attention for their potential to overcome these barriers (24). While numerous interleukins have been explored for their therapeutic potential in cancer immunotherapy, their clinical translation has often been constrained by untargeted activity and systemic toxicity. Among these, IL-12 remains one of the most extensively studied due to its capacity to promote antitumor immunity (25–27). However, despite promising pre-clinical results, early clinical trials of systemically administered recombinant IL-12 were limited by significant toxicity, leading to trial discontinuation and preventing a comprehensive evaluation of its therapeutic index and clinical efficacy in humans (28). These challenges have shifted the focus to developing novel delivery platforms that can localize IL-12 activity intratumorally and mitigate adverse effects.

**Figure 1:**
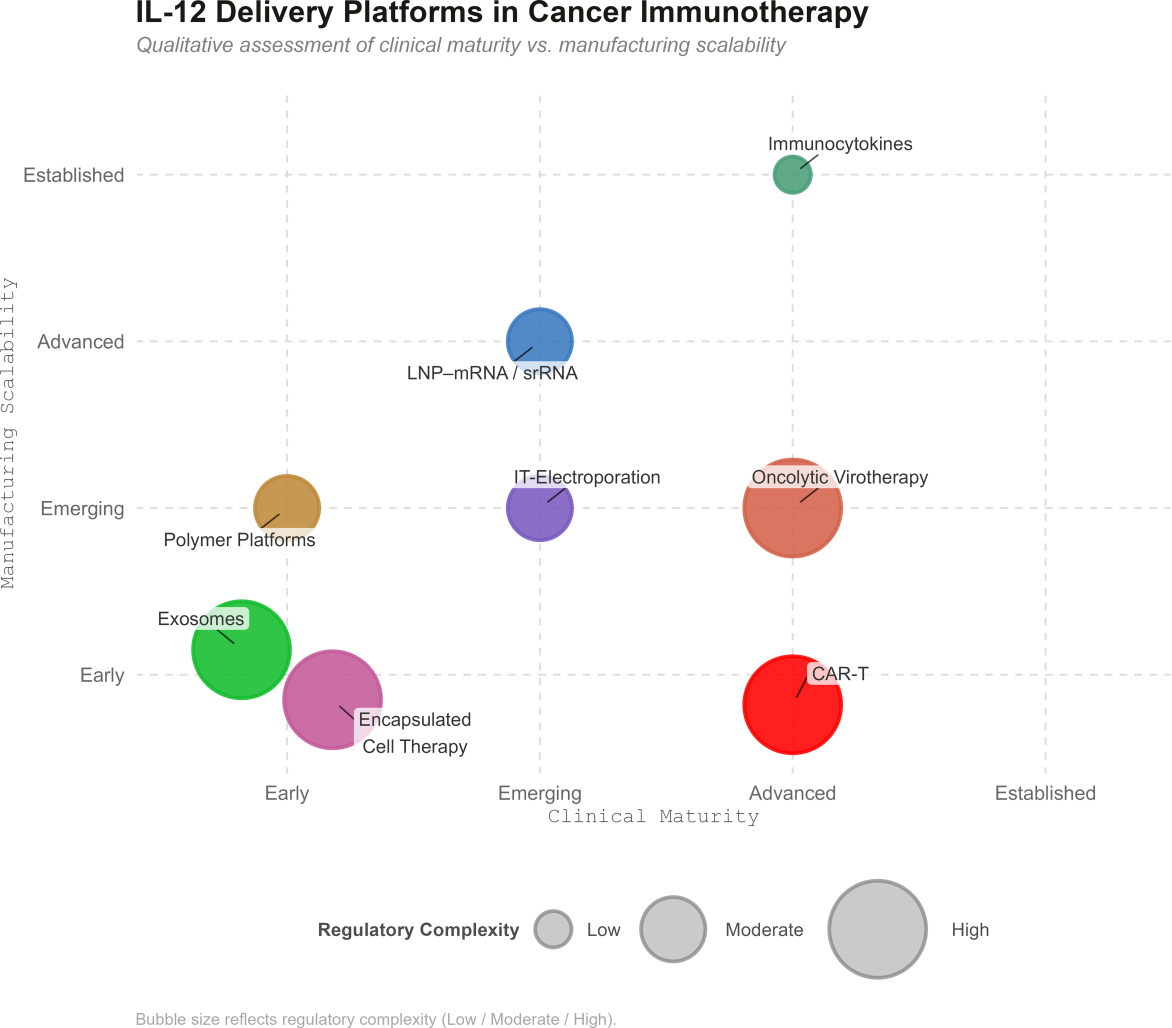
Comparative landscape of IL-12 delivery platforms for PDAC immunotherapy. Bubble plot comparing IL-12 delivery strategies across clinical maturity (*x*-axis) and manufacturing scalability (*y*-axis), scored 1–10 based on published trial data, regulatory precedent, and manufacturing feasibility. Bubble size reflects regulatory complexity (larger bubbles indicate higher complexity). Individual platform scores and rationale are provided in **Table 3**.

In this review, we will discuss existing and recent immunotherapies for PDAC, the rationale for developing IL-12-specific immunotherapy, the historical barriers to the systemic use of IL-12 therapies, and the most recent advances in delivery strategies (**Figure 1**) that aim to safely explore its potent immunostimulatory properties.

## 2 Pancreatic cancer immunotherapy

The aggressive nature and immunosuppressive microenvironment of PDAC often result in limited responses to traditional therapies. However, recent advancements in immunotherapy have opened new avenues for targeting this malignancy. In **Table 1**, we outline the primary immunotherapeutic approaches currently under investigation or in clinical use for PDAC.

**Table 1:** Immunotherapies for pancreatic cancer therapy.

Therapy	Mechanism	Key findings/challenges	Agent	Clinical trial
Targeting desmoplastic stroma	Modulates the tumor microenvironment (TME) by targeting the desmoplastic stroma to reduce immunosuppression	Mitigate immunosuppression in “cold” tumors, but effects are modest; TME complexity and dynamic shifts suggest multi-targeted approaches may be needed	PEGPH20 (hyaluronidase)	NCT02715804 NCT03481920
			FAK inhibitors (defactinib + pembrolizumab + chemotherapy)	NCT02546531
			CXCR4 inhibitors (BL-8040)	COMBAT trial
Adoptive cell transfer	Genetically or ex vivo –activated effector cells directed against tumor antigens or stress ligands	Better navigate the suppressive TME and engage cancer cells, but dense stroma in PDAC hinders infiltration and activity	TILs	NCT03610490
			TCR-T cells: KRAS G12D KRAS G12V	NCT03745326 NCT04146298
			CAR-T cells	NCT03323944 NCT01583686 NCT02159716 NCT07153289
			CAR-NK cells	NCT06816823 NCT00592293
			CIK-cells	NCT00965718 NCT05955157
Oncolytic virus	Enhances T-cell activity by blocking tumor immune evasion pathways	Overall response in PDAC remains low due to “cold” TME with low T-cell infiltration and high immunosuppressive cytokines	MEM 288 RGDCRAdcox2F	NCT05076760 NCT06693986
			Reovirus (Pelareorep)	NCT07280377
			HSV-based	NCT02446093
			LOAd703	NCT02705196 NCT03225989
Immune checkpoint inhibitors	Block inhibitory pathways exploited by cancer cells to restore T-cell activity	Limited efficacy as monotherapy in PDAC due to low immunogenicity and non-inflamed tumor phenotype	Anti PD-1/PD-L1	NCT02628067 NCT04802876
			Combination strategies	NCT07312422 NCT07208539 NCT04390399
Cancer vaccines	Stimulate immune response against PDAC-specific antigens	Dense stroma impairs efficacy; ongoing work focuses on identifying optimal antigens and delivery methods	KRAS targeted	NCT04853017 NCT05013216
			GVAX	NCT00084383 NCT02451982
			Personalized	NCT06496373 NCT03645148 NCT06156267

CAR, chimeric antigen receptor; FAK, focal adhesion kinase; NK, natural killer cell; PDAC, pancreatic ductal adenocarcinoma; TCR, T cell receptor; TIL, tumor-infiltrating lymphocyte; TME, tumor microenvironment.

Pancreatic cancer TME presents a complex barrier to effective immunotherapy, characterized by an extensive network of immunosuppressive mechanisms that render these tumors notoriously “cold” to immune-based interventions. The primary goal of immunotherapy is to target cells within the dense PDAC stroma or modulate the immune microenvironment, which could lead to “heat” the tumor [strategies summarized in (29)].

Several studies emphasize the need for strategies that target tumor cells and actively remodel the TME to facilitate immune cell infiltration and engagement, thereby potentiating immunotherapeutic outcomes in PDAC (**Table 1**). Moreover, the limitations of monotherapy, such as checkpoint inhibitors, are ineffective except in a rare subset of tumors due to the low immunogenicity and non-inflamed phenotype characteristic of PDAC.

The PDAC TME is dominated by pro-tumor immune cell populations, including myeloid-derived suppressor cells (MDSCs), M2-polarised tumor-associated macrophages (TAMs), N2 neutrophils, regulatory T cells (Tregs), and regulatory B cells, while simultaneously exhibiting a profound deficiency in antitumor effector cells such as CD8+ T cells, conventional dendritic cells, natural killer (NK) cells, and Th1 cells (30, 31). These immunosuppressive myeloid cells actively impair cytotoxic T-cell-mediated tumor destruction through multiple mechanisms, including the upregulation of PD-L1, nutrient depletion, and direct inhibition of dendritic cell function (32, 33).

Beyond cellular immunosuppression, PDAC is characterized by extensive desmoplasia, creating a dense fibrotic matrix that physically excludes immune effector cells from reaching tumor sites. Cancer-associated fibroblasts (CAFs) contribute to this hostile environment by secreting immunosuppressive factors, including IL-6, CXCL12, and GM-CSF (30). At the same time, the excessive extracellular matrix components limit drug penetration and CD8^+^ T cell infiltration. Additionally, the tumor-associated microbiome plays a crucial role in maintaining immunosuppression, with specific bacterial populations promoting M2 macrophage polarization and Th2 cell responses while simultaneously reducing antitumor Th1 and cytotoxic T cell activities (34–36).

Overall, this multifaceted immunosuppressive landscape explains why conventional immunotherapies, including immune checkpoint inhibitors, have shown limited efficacy in PDAC compared to other solid tumors. The convergence of cellular immunosuppression, stromal barriers, and microbial dysbiosis creates a self-reinforcing cycle of immune evasion that must be extensively addressed through combination therapeutic approaches targeting multiple components of this complex tumor environment.

## 3 Interleukin 12, immunotherapy, and PDAC

**Figure 2:**
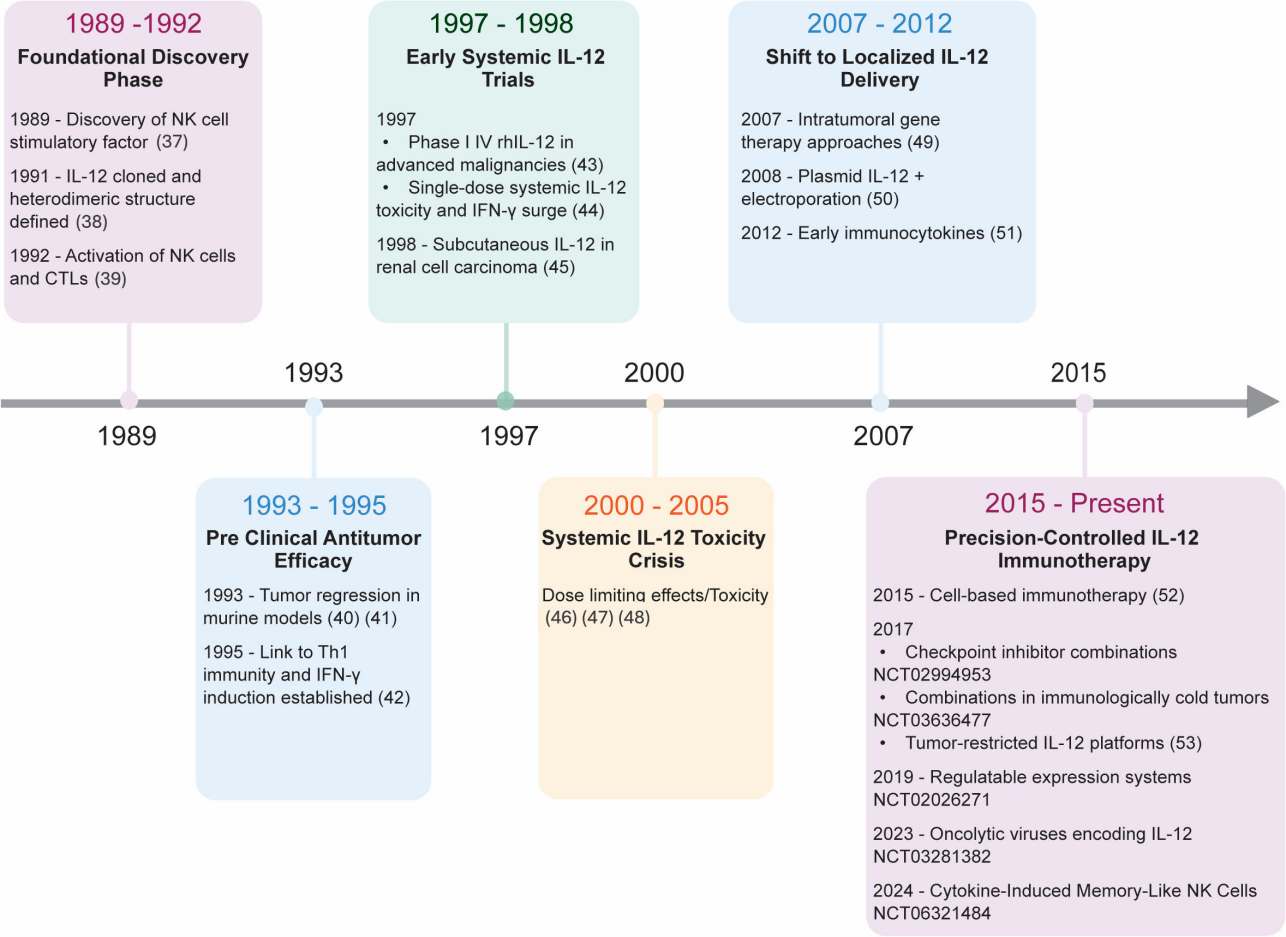
Chronological evolution of the therapeutic trajectory of IL-12 as cancer immunotherapy.

Interleukins are a family of secreted cytokines that have shaped cancer immunotherapy research since the 1970s, serving as both therapeutic targets and direct immunomodulatory agents. Among them, IL-12, originally described in 1989 as an NK cell stimulatory factor, has emerged as a particularly compelling candidate for tumors that resist conventional immune intervention (**Figure 2**). IL-12 is predominantly produced by antigen-presenting cells, including dendritic cells and macrophages, in response to tumor antigens and inflammatory stimuli (39). Its defining capacity to simultaneously engage innate and adaptive immunity distinguishes it from other cytokines in the immunotherapeutic armamentarium. These properties are especially relevant to PDAC, where, as described above, the convergence of cellular immunosuppression, stromal exclusion, and microbial dysbiosis has rendered conventional immunotherapy largely ineffective. This section articulates the mechanistic rationale for IL-12 as a therapeutic strategy in PDAC, situates it within the broader cytokine landscape, and discusses how localized delivery resolves the toxicity limitations that constrained earlier systemic approaches.

### 3.1 IL-12 mechanistically addresses PDAC-specific immune barriers

The immunosuppressive architecture of PDAC is composed of a layered set of reinforcing barriers, each of which IL-12 can disrupt through distinct and complementary mechanisms. The most fundamental barrier in PDAC is the near-complete exclusion of cytotoxic CD8+ T cells from the tumor parenchyma. This immune phenotype is maintained by the dominance of MDSCs and Tregs, which actively suppress T cell priming and function, and by the desmoplastic stroma, which physically prevents infiltration of effector cells. IL-12 addresses this exclusion at multiple levels. By driving the differentiation of naïve CD4+ T cells toward a Th1 phenotype and amplifying CTL and NK cell activation, IL-12 expands the pool of tumor-reactive effector cells available for recruitment (25, 54–56). Critically, IL-12-induced IFN-γ upregulates the chemokines CXCL9 and CXCL10, which promote T cell trafficking into the tumor bed, which is a mechanism that directly counteracts the chemokine exclusion maintained by MDSC and CAF activity in PDAC (57). Furthermore, preclinical studies demonstrate that IL-12 enhances granzyme B and perforin expression in CD8+ T cells, augmenting their cytotoxic capacity once they reach the tumor site (58, 59).

The myeloid dominance, specifically the abundance of M2-polarised tumor-associated macrophages and MDSCs that sustain immunosuppression through PD-L1 upregulation, nutrient depletion, and direct inhibition of dendritic cell function, is dominant in PDAC (32, 33). IL-12 directly reprograms this myeloid landscape by promoting macrophage polarization toward an M1 pro-inflammatory phenotype while suppressing M2-associated immunosuppressive functions (60). This macrophage repolarization is essential, as IL-12 converts immunosuppressive TAMs into tumor-hostile effectors, dismantling one of the central mechanisms of PDAC immune evasion (61, 62). Similarly, IL-12 has been shown to reduce MDSC concentration and suppress Treg function in the TME, further relieving the suppressive pressure on infiltrating effector cells (63, 64).

PDAC desmoplastic stroma, composed of a dense fibrotic matrix secreted principally by CAFs, represents a structurally distinctive immune barrier that distinguishes this tumor from most other solid tumors where immunotherapy has succeeded. IL-12, through sustained IFN-γ signaling, has been shown to modulate fibroblast activation and reduce the secretion of immunosuppressive stromal factors, potentially rendering the stroma more permissive to immune cell penetration (65, 66). This stromal remodeling capacity is absent from most other cytokine candidates and represents a mechanistic advantage specific to IL-12 downstream signaling program.

Finally, PDAC is characterized by profound tumor hypoxia, which further suppresses immune function and promotes angiogenesis via VEGF upregulation. IL-12 anti-angiogenic activity, mediated through CXCL10-dependent suppression of neovascularization (67), offers an additional mechanism relevant to this feature of the PDAC microenvironment. By limiting tumor vascularization, IL-12 may simultaneously reduce the hypoxia-driven immune escape and improve drug penetration within the desmoplastic tumor core.

Localized IL-12 mRNA delivery in preclinical PDAC models reinvigorated exhausted tumor-infiltrating T cells and reduced metastatic burden, demonstrating local-to-systemic propagation of antitumor immunity consistent with the mechanisms described above (64). These findings anchor the broader mechanistic argument in PDAC-specific biology and provide proof of concept for the delivery strategies discussed in subsequent sections.

### 3.2 Advantages of IL-12 over other cytokines

Although several cytokines can remodulate the tumor immune environment, the immunological context of PDAC imposes specific limitations on cytokine selection.

IL-2, the earliest clinically deployed cytokine immunotherapy, promotes T and NK cell proliferation but preferentially expands Foxp3+ regulatory T cells at therapeutic doses, which hinders its use, particularly in PDAC, where Tregs already dominate the TME, and further Treg expansion would reinforce rather than disrupt immune exclusion (26, 68). IL-15 has similar signaling components as IL-2 but has a lower propensity for Treg expansion, supporting T and NK cell survival and proliferation but lacks IL-12 capacity to drive Th1 polarization, reprogram myeloid cells, or remodel the stromal compartment (69). As discussed above, these are functions that are specifically required to address PDAC layered immune barriers. IL-18, which synergizes with IL-12 to amplify IFN-γ production, is insufficient as a monotherapy in cold tumors and requires IL-12 co-stimulation to achieve meaningful antitumor activity, underscoring IL-12 role as the upstream orchestrator of this cytokine axis (70). Immunoregulatory cytokines such as IL-10 and IL-6, while relevant to PDAC biology as CAF-secreted immunosuppressive mediators (30), can exert tumor-promoting effects depending on the context, and are not appropriate immunotherapeutic candidates in this setting. By contrast, IL-12 uniquely coordinates both innate and adaptive immune activation through a single signaling axis of IL-12-IFN-γ-Th1 circuit, providing a broad signaling of downstream effects that no single alternative cytokine recapitulates.

### 3.3 Synergy with immune checkpoint inhibitors and combinatorial strategies

IL-12 further distinguishes itself through its capacity to interface with immune checkpoint pathways that are central to PDAC immune evasion. Checkpoint molecules, including PD-1 and CTLA-4, contribute to T cell dysfunction within the PDAC TME (71, 72). Single-agent checkpoint blockade has shown minimal efficacy in unselected PDAC (73), in large part because the T cell exclusion remains unaddressed with this therapy. IL-12 targets this upstream barrier directly, expanding and recruiting cytotoxic T cells into the tumor before checkpoint reinvigoration is attempted. Strategies combining IL-12 with PD-L1 blockade have demonstrated synergistic antitumor effects and prolonged survival in preclinical models (74–76), consistent with the concept that IL-12 resolves immune exclusion while checkpoint inhibitors restore effector function in cells already present within the tumor. This mechanistic complementarity positions IL-12 not as an alternative to checkpoint therapy but as an enabling combination partner that addresses PDAC primary resistance mechanism.

Beyond checkpoint inhibition, IL-12 has demonstrated synergy with other cytokines and immunomodulatory agents. Combined delivery of IL-7 and IL-12 has been shown to drive tumor regression and facilitate systemic antitumor responses by activating tumor-infiltrating lymphocytes, rendering distant tumors sensitive to checkpoint blockade (77). IL-12 co-expression within CAR-T cell constructs represents an emerging combinatorial strategy, wherein locally secreted IL-12 remodels the suppressive PDAC stroma to sustain CAR-T cell function (78). Collectively, these findings position IL-12 as a versatile immunological amplifier within multimodal therapeutic frameworks, with particular relevance to the combinatorial approaches that PDAC complex TME demands.

### 3.4 Localized delivery as the resolution for systemic toxicity

Despite IL-12 potent immunostimulatory profile, early clinical trials of systemic recombinant IL-12 yielded minimal therapeutic benefit due to dose-limiting toxicity (28, 43, 44). Mechanistically, supraphysiologic serum IL-12 concentrations activate circulating lymphocytes and NK cells indiscriminately, triggering uncontrolled IFN-γ release and downstream sequelae, including cytokine release syndrome, hepatotoxicity, and vascular injury (28, 43, 44, 79–81). These toxicities arise from systemic immune activation rather than antitumor immune priming, and are therefore a function of delivery route rather than an intrinsic limitation of IL-12 biology.

Localized IL-12 delivery within the PDAC TME preserves the immunostimulatory mechanisms described above while confining their activation to the tumor and its draining lymph nodes. Intratumoral IL-12 promotes dendritic cell maturation and antigen uptake, facilitating lymph node migration and priming of tumor-specific CD8+ T cells (82). These effector cells subsequently traffic systemically, mediating immune surveillance at distant sites, which is an abscopal mechanism documented in preclinical PDAC models, in which localized IL-12 mRNA reduced metastatic burden without elevating serum IL-12 or IFN-γ to toxic levels (64, 81, 83). This local-to-systemic immune propagation provides a mechanistic basis for how tumor-confined IL-12 activity can elicit durable systemic antitumor responses while avoiding the cytokine storm associated with systemic administration.

Taken together, the mechanistic properties of IL-12, including spanning Th1 polarization, CTL and NK activation, myeloid reprogramming, stromal modulation, anti-angiogenesis, and checkpoint synergy, align directly and specifically onto the immune barriers that define PDAC therapeutic resistance (**Table 2**). No alternative cytokine candidate addresses this full spectrum of PDAC-relevant mechanisms through a single signaling axis. In PDAC, where immune exclusion, myeloid dominance, and desmoplastic stroma collectively enforce a self-reinforcing cycle of immune evasion, localized IL-12 delivery offers a mechanistically grounded strategy to reshape the tumor immune landscape, enable combination immunotherapy, and elicit systemic antitumor responses with a manageable safety profile. The delivery platforms for achieving this localization are discussed in the following sections.

**Table 2:** Main immunomodulatory effects of IL-12.

Main effect	Mechanism	Outcome in cancer immunotherapy	Ref
Activation of T cells and NK cells	Promotes proliferation and cytolytic maturation of T cells and NK cells, enhancing their cytotoxic activity	Increased tumor cell killing by activated immune effectors	(84, 85)
Induction of IFN-γ production	Stimulates T cells and NK cells to produce IFN-γ, leading to downstream effects like increased MHC expression and enhanced antigen presentation	Sustained inflammation and improved immune recognition of tumor cells	(54, 56)
Anti-angiogenesis	Induces chemokines such as CXCL10/IP-10 via IFN-γ, thereby inhibiting tumor blood vessel formation	Reduced tumor vascularization, limiting tumor growth and metastasis	(66, 86, 87)
Tumor microenvironment remodeling	Reduces immunosuppressive cells and polarizes macrophages from M2 to M1 phenotype	Reversal of tumor-supporting immunosuppression, enhancing overall antitumor immunity	(27, 61, 62)
Induction of immune memory	Promotes the generation of long-lasting memory T cells	Provides durable protection against tumor recurrence	(25, 55)

IFN-γ, interferon gamma; MHC, major histocompatibility complex; NK, natural killer cell.

## 4 Interleukin 12 delivery systems

The delivery systems for IL-12 have evolved to enhance its therapeutic potential while minimizing systemic toxicity, a significant drawback observed in early studies in which systemic administration of recombinant IL-12 led to severe adverse events (88, 89). Intratumoral delivery strategies have been developed to increase intratumoral concentration and enhance the recruitment and activation of various immune effector cells while tolerating lower doses to mitigate toxic effects. These innovations, particularly from 2015 to the present, are summarized in **Table 3** and have demonstrated efficacy in preclinical models, showcasing IL-12’s ability to reverse tumor-induced immunosuppression, as discussed below.

**Table 3:** Comparison of delivery strategies for IL-12-based immunotherapy in PDAC.

Strategy	Delivery route	Spatial control	Duration of IL-12 expression	Controllability/Reversibility	Systemic leakage risk	CRS/Toxicity risk	Stromal barrier penetration	Reversal of immune exclusion	Myeloid/Treg modulation	Key limitations
Immunocytokines	IV or locoregional	Low–moderate	Short (hours–days)	Limited (dose-dependent only)	High	Moderate–high	Limited in dense stroma	Moderate	Moderate	Systemic toxicity; short half-life; poor stromal penetration
LNP–mRNA/srRNA	IV or intratumoral	High (IT); Low (IV)	Days (mRNA); weeks (srRNA)	Moderate (dose, repeat dosing)	Low (IT); High (IV)	Moderate	Limited (IV), better if IT	Potentially strong if sufficient expression	High	Transient expression; LNP immunogenicity; cold chain
IT-Electroporation	Intratumoral (EUS-guided)	High	Transient (days–weeks)	Limited once transfected	Low	Low	Poor (restricted to accessible lesions)	Variable (inconsistent abscopal effect)	High	Procedure-bound; superficial access required; unsuitable for diffuse metastases
CAR-T (IL-12 armored)	IV or locoregional	Low–moderate	Sustained (weeks–months)	Low (unless suicide switch present)	Moderate	High	Limited in solid tumors	Potentially strong but inconsistent	High	Stromal exclusion; CRS risk; autologous manufacturing bottleneck; T cell exhaustion
Oncolytic Virotherapy (IL-12-armed)	Intratumoral or IV	Moderate	Variable (replication-dependent)	Limited (once replicating)	Low–moderate	Moderate	Moderate (replicative spread)	Strong (viral inflammation)	High	Anti-viral immunity limits re-dosing; poor desmoplastic penetration; GMO regulation
Polymer Platforms	Intratumoral depot	High	Tunable (days–weeks)	Moderate (design-dependent)	Low	Low	Limited beyond depot radius	Moderate	Moderate–high	Desmoplastic stromal barrier; manufacture challenges; largely preclinical
Encapsulated Cell Therapy	Locoregional implant	High	Long (weeks–months)	Moderate (implant removal possible)	Low	Low	Limited to implantation site	Moderate	Moderate	Capsule fibrosis; limited IL-12 diffusion; surgical implantation; cell viability
Exosomes (IL-12 or mRNA cargo)	IV or intratumoral	Moderate	Short (days)	Moderate (repeat dosing)	Low–moderate	Low	Better than large biologics	Potentially strong	Moderate	Low yield; batch variability; no EV regulatory framework; short half-life

EUS, endoscopic ultrasound; IL-12, interleukin 12; IT, intratumoral; IV, intravenous; SC, subcutaneous; TME, tumor microenvironment; Treg, T regulatory cell.

### 4.1 Systemic administration of recombinant IL-12

Following promising results from pre-clinical and animal studies, early clinical trials in the mid-90s began investigating systemic IL-12 administration in patients with various cancer types (90). Initial Phase I dose-escalation studies of recombinant human IL-12 aimed to assess toxicity, identify the maximum tolerated dose (MTD), and evaluate biological activity in patients with advanced cancer (43, 90, 91).

Following this pilot study, a subsequent larger Phase I clinical trial administered IL-12 intravenously for five consecutive days in repeated cycles to patients with renal cell carcinoma, melanoma, and colon cancer. This study identified an MTD of 500 ng/kg, with manageable toxicities and evidence of strong immune activation (43, 90). However, a follow-up Phase II trial in patients with renal cancer modified the dosing regimen by omitting the initial preceding low-dose priming injection used in the previous study. Administering high-dose treatment immediately resulted in severe systemic toxicity, including two patient deaths, which led to the early termination of the trial (44, 90). Subsequent analyses suggested that IL-12 toxicity is strongly dependent on the dosing regimen, particularly the inclusion of an initial low-dose priming injection (92, 93). The priming dose in the Phase I trial likely conditioned the immune system, reducing the intensity of cytokine release during the subsequent high-dose administrations and improving tolerability. In contrast, the Phase II trial started directly with high doses, leading to an uncontrolled immune response and severe toxicity, which resulted in early trial termination (44, 90, 92, 93). These results highlight the importance of dosing strategy in minimizing IL-12–related toxicity.

In the following months, IL-12 trials resumed at several centers using various systemic delivery routes, intravenous (46, 94, 95), subcutaneous (45, 96–98), and intraperitoneal (80) and tested a range of escalating doses and treatment schedules (80). A comprehensive summary of these trials is provided in a recent review (90). Most trials established MTDs between 250–500 ng/kg, with the more intensive regimens incorporating an initial priming injection to mitigate toxicity (80, 98). Despite reduced adverse effects, this approach did not improve the overall therapeutic efficacy of IL-12 (80).

A fundamental limitation of systemic administration of recombinant IL-12 therapy is its poor tumor targeting. The systemic distribution of the cytokine tends to yield strong off-target effects with systemic activation and proliferation of immune cells, making it difficult to achieve adequate intratumoral therapeutic levels without exceeding systemic toxicity thresholds (80, 99). Common toxicities reported across trials include nausea, vomiting, fatigue, fever, flu-like symptoms, diarrhea, headaches, cytokine-release syndrome, and hepatic toxicities (43, 80, 90, 96, 98).

Taken together, systemic administration of IL-12 allows whole-body exposure, and early clinical trials revealed severe toxicities, which were only partially alleviated by adjusting the dose and treatment schedule. Despite these modifications, patient outcomes remained modest, with disappointing antitumor efficacy. This ineffectiveness is likely due to the inability of systemically delivered IL-12 to achieve therapeutically effective concentrations within the tumor microenvironment at the maximum tolerated dose. Consequently, systemic IL-12 therapy has been halted due to the modest positive effects and the widespread off-target toxicity. Although most of these early systemic IL-12 trials were conducted in melanoma, renal cell carcinoma, and colon cancer, the knowledge gained of IL-12 immunomodulatory characteristics is highly relevant to the challenges faced in PDAC, where immunotherapy has similarly struggled due to poor tumor penetration and systemic toxicity. To overcome the systemic diffusion of IL-12, recent studies have developed novel delivery strategies to more effectively localize IL-12 activity at the tumor site while minimizing systemic toxicity. These approaches include virotherapy, such as retrovirus- and adenovirus-mediated delivery, lipid nanoparticle encapsulation of IL-12 mRNA, and polymer-encapsulated engineered cytokine-secreting cells.

### 4.2 Virotherapy

Virotherapy has emerged as a promising alternative therapeutic approach for malignancies such as pancreatic cancer. Various viral platforms, including oncolytic adenoviruses, helper-dependent adenoviruses, and retroviruses, have been engineered to deliver therapeutic genes directly to tumors, enabling localized treatment while minimizing systemic toxicities (100–102). Increasingly, these vectors are used to provide immunostimulatory genes that can enhance antitumor immunity without requiring widespread viral replication, with IL-12 being a focus (103, 104). These viruses are designed to infect tumor cells selectively, allowing high local concentrations of IL-12 to stimulate immune cell infiltration and tumor regression while limiting systemic exposure (100, 101). By combining targeted delivery with localized cytokine expression, virotherapy aims to maximize antitumor efficacy and safety in pancreatic cancer models.

A conditionally replicative adenovirus (CRAd) designed to selectively replicate in tumor cells exploits hypoxia-inducible factor activity and the dysfunction of the retinoblastoma pathway (100). This virus, termed Ad-DHscIL12, was engineered to express IL-12 and tested in an immunocompetent Syrian hamster model of pancreatic cancer. A single intratumoral injection induced robust IL-12 expression and potent antitumor activity, yielding greater therapeutic efficacy than replication-competent luciferase control viruses, which required higher doses to achieve comparatively modest effects. The study also reported increased CD3^+^ T-cell infiltration in tumors and low circulating IL-12 levels, indicating localized immune activation with minimal systemic toxicity. Furthermore, the antitumor response was durable, persisting for several weeks after treatment. These findings support the potential of a tumor-restricted CRAd platform as a localized, safer, and more efficacious modality for IL-12-based gene therapy in pancreatic cancer (100).

In 2015, two adenoviral gene therapy platforms for IL-12 delivery were compared in an orthotopic PDAC model with liver metastases in immunocompetent Syrian hamsters (101). They evaluated novel oncolytic adenoviruses (OAds) encoding membrane-anchored single-chain IL-12 (scIL-12) variants alongside a previously developed helper-dependent adenovirus (HDAd) containing a liver-specific, mifepristone-inducible IL-12 expression system (HC-Ad/RUmIL-12). Both vectors induced strong antitumor responses; however, OAds expressing IL-12 led to acute systemic toxicities due to transiently elevated serum IL-12 levels, even at subtherapeutic doses. To mitigate this, IL-12 variants were anchored to the cell membrane, restricting cytokine activity to the tumor microenvironment, which substantially reduced systemic exposure. Although this safety improvement was achieved, it moderately reduced therapeutic efficacy. In contrast, the HDAd vector with a mifepristone-inducible promoter enabled tightly regulated IL-12 expression in hepatocytes, effectively inhibiting liver metastases with minimal toxicity. Overall, the study highlighted the contrasting efficacy and safety between local and systemic IL-12 delivery approaches, with HDAds emerging as a precise and better-tolerated strategy for cytokine-based immunotherapy in PDAC (101).

An innovative approach, motivated by previous findings that even membrane-anchored IL-12 can saturate the cell surface and subsequently release IL-12 into circulation, causing systemic toxicity (101), was introduced to reduce IL-12-associated toxicity without compromising its antitumor efficacy (103). To address this, researchers engineered an IL-12 variant lacking the N-terminal signal peptide, leading to intracellular retention of the cytokine. This IL-12 variant was delivered via a tumor-selective oncolytic adenovirus (Ad-TD) in immunocompetent Syrian hamster models of PDAC. Intraperitoneal administration significantly prolonged survival in animals bearing orthotopic tumors and induced complete regression of peritoneal metastases, all without detectable systemic toxicity, unlike the outcomes observed with unmodified IL-12. These results support the use of intracellular cytokine retention as a promising strategy to enhance the safety and therapeutic efficacy of IL-12-based gene therapies for pancreatic cancer (103).

The delivery of IL-12 via adenoviruses has also been explored in combination approaches to enhance antitumor immunity. An adenoviral vector was used to co-deliver IL-12 and the co-stimulatory molecule B7.1 in a non-immunogenic murine PDAC model (104). This strategy achieved complete tumor regression in 80% of treated mice and elicited prolonged immune memory after a single intratumoral injection. Upon rechallenge with parental tumor cells, 70% of cured animals remained tumor-free, demonstrating durable protective antitumor immunity. These findings highlight the potential of combinatorial IL-12 gene therapy strategies to achieve tumor eradication and long-term immunological memory in PDAC (104).

Retroviral delivery systems have also been investigated for IL-12 gene therapy. AsPC-1 pancreatic tumor cells were engineered via retroviral transduction to express IL-12 (102). Subcutaneous implantation of these cells into mice resulted in significantly reduced tumor growth and prolonged survival compared to controls, without systemic toxicity or weight loss being observed. These results highlight the therapeutic potential of tumor-targeted IL-12 gene delivery for suppressing tumor progression and extending survival while minimizing adverse effects in murine pancreatic cancer models (102).

Overall, these studies demonstrate that tumor-localized IL-12 delivery via oncolytic adenoviruses or retroviral vectors is an effective strategy to achieve potent antitumor responses while minimizing systemic toxicity. Strategies such as membrane-anchored or intracellularly retained IL-12, inducible promoters, and combination with co-stimulatory molecules like B7.1 have enhanced local immune activation, increased T-cell infiltration, reduced systemic IL-12 diffusion, and promoted tumor regression. Furthermore, these approaches provide durable antitumor immunity and prolonged survival across multiple pancreatic cancer models, highlighting their translational potential as a targeted, well-tolerated strategy for IL-12-based cancer therapy. These pre-clinical studies have supported the initiation of early-phase clinical trials evaluating IL-12 virotherapy in cancer patients (NCT05538624, NCT03281382).

#### 4.2.1 Virotherapy in clinical trials for PDAC

Clinical translation of IL-12-based virotherapy for pancreatic cancer is still in its early stages, with only limited clinical investigations completed to date. The most notable work consists of a Phase I trial and subsequent long-term follow-up evaluating an oncolytic adenoviral platform co-expressing IL-12 in patients with metastatic PDAC (105, 106).

Between 2017 and 2019, a Phase I dose-escalation trial evaluated the safety and feasibility of intratumoral injection of an oncolytic adenoviral vector co-expressing IL-12 and suicide genes in patients with metastatic PDAC. This study represented the first clinical application of IL-12 immunogene therapy combined with an oncolytic adenoviral suicide gene platform for the treatment of PDAC. This platform, previously tested in prostate cancer (107–109), incorporates yeast cytosine deaminase and a mutant herpes simplex virus thymidine kinase suicide gene to convert administered prodrugs into local cytotoxic metabolites, potentially enhancing tumor cell killing while stimulating immune activation. To augment these effects, the vector was engineered to co-express IL-12, thereby stimulating antitumor immunity by activating NK cells and cytotoxic T lymphocytes. Clinically, twelve patients with liver-metastatic PDAC received a single intratumoral injection at three dose levels, followed by prodrug 5-fluorocytosine, and in some cases standard chemotherapy regimens (FOLFIRINOX or gemcitabine/nab-paclitaxel) 21 days later. The treatment was well tolerated, with only one dose-limiting toxicity observed at the highest dose. As a result, the maximum tolerated dose was not reached, and no serious adverse events occurred in five of six patients at the highest dose. Notably, four out of six patients in the highest dose cohort were still alive at follow-up in 2020, while all patients in the lower-dose groups succumbed to disease progression. These findings support the therapeutic potential of oncolytic adenoviral vectors for localized IL-12 delivery, as well as the combination of immune-stimulatory cytokine gene therapy with tumor-selective cytotoxicity in PDAC (106).

A 2024 long-term follow-up study confirmed a dose-dependent survival benefit from the previous original Phase I trial. Patients in the highest-dose group had a median overall survival of 18.4 months, markedly longer than 4.8 months and 3.5 months in the intermediate and lowest-dose groups, respectively. Notably, one patient in the highest-dose cohort achieved an overall survival of 59.1 months with complete radiographic responses in liver and lung metastases. These findings further support IL-12 and suicide gene therapy’s clinical potential in metastatic PDAC, particularly at higher therapeutic doses (105).

Collectively, these findings demonstrate that oncolytic virotherapy incorporating IL-12 is a clinically viable and well-tolerated strategy at therapeutic doses, capable of eliciting durable responses in a subset of patients with advanced PDAC. Although current evidence is limited to small, early-phase studies, the observed dose-dependent survival benefits, including a complete radiographic response in one patient, highlight the clinical potential of this approach (106). Continued clinical evaluation in larger, controlled trials will be essential to refine dosing strategies and define its role in combination with other treatment methods for pancreatic cancer.

In summary, while oncolytic virotherapy represents a promising platform for IL-12 delivery, enabling localized cytokine release to potentiate antitumor immunity, its clinical advancement is tempered by several critical limitations, including the potent immunostimulatory activity of IL-12, which risks systemic toxicity through cytokine release syndrome and inflammatory cascades (77, 101). Additionally, the immunosuppressive TME often blunts interleukin’s capacity to activate T cells and drive regression, exacerbated by interpatient heterogeneity and evolving tumor resistance mechanisms, such as downregulated checkpoint pathways, which underscore the unpredictability of responses and the imperative for adaptive therapeutic monitoring (110). The immunogenicity of oncolytic viruses poses notable challenges due to the presence of neutralizing antibodies (NAbs) in a significant portion of the human population, with potential implications for the efficacy of oncolytic virotherapy (111–113). These NAbs can severely compromise therapeutic efficacy by blocking viral infection and replication, limiting both initial treatment responses and repeat-dosing strategies, as demonstrated in clinical trials in which anti-adenoviral antibodies correlated with reduced survival in glioma patients (114, 115). Despite the challenges posed by neutralizing antibodies, antiviral immunity may paradoxically enhance therapeutic outcomes in specific contexts in which pre-existing immunity to oncolytic viruses potentiates systemic antitumor immunity, suggesting that controlled immune responses against the virus can amplify the desired anticancer immune activation (113). The combination of these hurdles highlights the need for a comprehensive understanding and careful management of such therapies.

### 4.3 Polymer-encapsulated engineered cytokine-secreting cells

Cell-based immunotherapies, including adoptive transfer of tumor-infiltrating lymphocytes (TILs), chimeric antigen receptor (CAR) T cells expressing IL-12, have potent antitumor activity in preclinical models and early clinical trials. However, these approaches are often associated with complex manufacturing, limited control over *in vivo* cytokine release, and risks of systemic toxicity (81, 116). Encapsulation-based delivery addresses these limitations by providing spatial confinement of IL-12 production, preventing systemic cytokine distribution while maintaining localized immunostimulatory activity, and by eliminating the need for complex autologous cell engineering and patient-specific manufacturing. Encapsulation emerged prominently in the past decade to overcome the limitations of systemic IL-12 administration. To mitigate systemic and off-target effects of interleukin therapies, these recent innovations encapsulate genetically engineered cytokine-secreting cells within biocompatible matrices, such as alginate or pH-sensitive polymers. Although no IL-12 encapsulation therapies have entered clinical trials this year, an ongoing Phase I evaluation of alginate-encapsulated IL-2-secreting cells for ovarian cancer (AVB-001) is underway, following success in pre-clinical murine models (NCT05538624). These results unlock the possibility of IL-12’s full therapeutic potential in refractory solid tumors, such as PDAC.

In a proof-of-concept study, alginate-microencapsulated NIH3T3 fibroblasts engineered to constitutively secrete murine IL-12 were implanted peritumorally in a syngeneic CT26 colon carcinoma model to achieve sustained, localized cytokine delivery (117). The semipermeable alginate microcapsules permitted continuous diffusion of bioactive IL-12 while physically isolating the engineered cells from host immune rejection, enabling long-term cytokine release *in vivo*. Mice receiving IL-12–secreting microcapsules exhibited marked inhibition of tumor growth, with mean tumor volumes reduced by over 70–80% compared with control animals by day 21 post-implantation, and a significant delay in tumor progression. These effects were accompanied by prolonged survival, with median survival extended from ~25 days in controls to ~45–60 days in the IL-12–treated cohorts, and a subset of animals achieving complete tumor regression. Localized IL-12 production elicited robust systemic immune activation, characterized by elevated serum levels of Th1-associated cytokines, including IL-12, IL-2, and IFN-γ, and by enhanced activation of natural killer (NK) cells and cytotoxic T lymphocytes (CTLs). Importantly, despite this potent systemic antitumor immunity, treated animals did not exhibit the severe toxicities typically associated with systemic administration of recombinant IL-12, underscoring that therapeutic efficacy was achieved through immune cell–mediated propagation rather than high circulating cytokine levels (117).

In a 2022 study, a cytokine delivery platform was developed using polymer-encapsulated, genetically modified human retinal pigmented epithelial (RPE) cells (118). These cells were engineered using the PiggyBac transposon system to stably express the natural cytokine IL-2. The IL-2-secreting RPE cells were then encapsulated within a biocompatible polymer scaffold (polymer-encapsulated) and implanted in preclinical mouse models of ovarian and colorectal cancer to achieve localized cytokine delivery. Following implantation, the encapsulated RPE cells remained viable, non-proliferative, and continuously secreted IL-2, persisting *in vivo* longer than unencapsulated controls (118). Intraperitoneal IL-2 concentrations exceeded systemic (blood) levels by more than 100-fold, demonstrating that local cytokine levels can be tightly controlled. IL-2 concentrations peaked after 24 hours and remained above the threshold required for effector T-cell binding for over 14 days, until the capsule’s surface was covered with pericapsular fibrotic overgrowth, which halted cytokine delivery (118). Importantly, this localized delivery eradicated peritoneal tumors in both colorectal and ovarian cancer mouse models without inducing observable systemic toxicity (118).

In a 2025 follow-up study, the same polymer-encapsulation platform was applied to deliver IL-12 using genetically modified human RPE cells (61). The IL-12-secreting RPE cells were encapsulated within a biocompatible polymer scaffold and implanted into pre-clinical mouse models representing a range of tumor types, including pancreatic cancer (61). After just 5 days of implantation, mice bearing pancreatic tumors exhibited significantly fewer viable tumor cells than control animals, demonstrating the rapid ability of these cytokine factories to activate antitumor immune responses. Furthermore, with continued IL-12 treatment for 120 days, complete elimination of pancreatic tumors was observed, with no evidence of weight loss or systemic toxicity. Notably, the antitumor response was mediated primarily by T cells and inflammatory monocytes, in contrast to the IL-2-based platform, which predominantly activated NK cells to induce antitumor responses. Taken together, these findings demonstrate that encapsulated IL-12-secreting RPE cells can safely and effectively drive potent antitumor immunity and eliminate tumors, significantly prolonging survival in PDAC-bearing mice while avoiding the systemic toxicities and off-target effects typically associated with other IL-12 therapies.

These studies demonstrate the therapeutic potential of encapsulated cytokine-secreting cells as a versatile, controllable immunotherapy platform that can improve the pharmacokinetic profiles of interleukins while also enhancing their clinical efficacy as cancer treatments.

### 4.4 Nanoparticles-based delivery

#### 4.4.1 Lipid nanoparticle encapsulation of mRNA

mRNA cytokine therapy has been shown to extend cytokine half-life, reduce production costs, and exhibit fewer toxicities compared to systemic administration of recombinant IL-12. While mRNA therapies are typically delivered via intratumoral injection, in cancers like PDAC with visceral tumors, direct intratumoral delivery requires invasive procedures, which carry risks of complications and may be technically challenging for repeated administrations (119, 120). To circumvent the invasiveness of direct intratumoral injection, a noninvasive method to deliver IL-12 to PDAC tissues via intraperitoneal injection was developed using lipid nanoparticles composed of an ionizable lipid, a phospholipid, cholesterol, and a PEG-conjugated lipid, at a ratio of 16:8:8:3, optimized for mRNA delivery efficiency and cellular uptake in pancreatic tissues (119). Using an orthotopic mouse model, a single intraperitoneal injection of pancreas-targeting lipid nanoparticles that delivered IL-12 mRNA remodeled the tumor microenvironment. There was increased activation of antigen-presenting cells, NK cells, and CD8+ T cells, indicating a robust antitumor immune response. Furthermore, significant tumor regression and increased survival were observed in treated mice, with some mice achieving complete tumor elimination. The mice tolerated the treatment well, with minimal changes in body weight and no significant toxicities observed. Taken together, these results demonstrate that the nanoparticles successfully delivered mRNA-based IL-12 therapy to PDAC tissues, thereby improving PDAC responsiveness to immunotherapy in a safe, noninvasive, and targeted manner in mice (119).

IL-12 has also been delivered to PDAC tissues using biodegradable polylactic acid microspheres. The efficiency of this IL-12 delivery mode was explored in combination with stereotactic body radiation therapy (SBRT) in pre-clinical murine models of PDAC (62). Biodegradable polylactic acid microspheres were used to encapsulate recombinant IL-12, following methods developed in earlier studies that demonstrated effective local and sustained cytokine delivery to induce antitumor responses in mice bearing lung alveolar cell carcinoma tumors (121, 122). A comparison of injection methods revealed that microspheres injected intratumorally were sequestered within the tumor, whereas those injected intraperitoneally were trafficked into the bloodstream. This demonstrates that, in addition to the encapsulation technology used, the mode of administration also plays a significant role in the efficiency and outcome of IL-12 treatment. Polymer microspheres encapsulating recombinant IL-12 protein were intratumorally injected 24 hours after SBRT treatment in PDAC mouse models. The results showed that local IL-12 delivery, combined with SBRT, led to tumor eradication by day 20 after microsphere administration in mice with KCKO-luc tumors. The combination therapy also resulted in 100% of mice achieving long-term survival. These findings were replicated in mice bearing Pan02 tumors, in which increased long-term survival and reduced tumor burden were also observed. Taken together, these results demonstrate the efficiency of this technology in delivering local IL-12 and inducing effective antitumor responses in pre-clinical murine PDAC models (62).

A follow-up study used a unique IL-12 mRNA sequence in which the p35 and p40 heterodimeric subunits were fused via a polypeptide linker and incorporated a miR-122 binding domain, which limits off-target translation in the liver (64). Building on their previous study, IL-12 mRNA was encapsulated in the same lipid nanoparticle technology, which had previously been shown to be safe and effective for delivering local cytokine therapy to induce antitumor activity. Intratumoral delivery of IL-12 encapsulated in lipid nanoparticles resulted in a shift from an immunosuppressive to an immunostimulatory tumor microenvironment, with T cells mitigating their exhausted phenotype, and increased T-cell activation and proliferation were observed. Taken together, the authors conclude that this method outperformed the method used in their previous study (62). When IL-12 concentrations were quantified after intratumoral administration, they peaked at 24 hours and returned to near baseline levels by 96 hours. Concentrations were similar in the pancreatic draining lymph node; however, distal tissues, such as the liver and non-draining lymph nodes, had approximately 20-fold lower levels of IL-12 at 24 hours. The blood levels of liver and kidney functional markers also remained within normal physiological ranges after IL-12 treatment, demonstrating that IL-12 was concentrated locally and did not induce liver or kidney toxicities. When local delivery of encapsulated IL-12 via intratumoral injection and SBRT were combined, it resulted in a dramatic reduction in primary tumor burden and increased long-term survival in multiple murine PDAC models (64). Taken together, the encapsulation of IL-12 mRNA in lipid nanoparticles has been shown to be safe and effective in inducing local antitumor effects against PDAC tumors (64).

Beyond conventional mRNA delivery, recent studies have explored self-replicating RNA (srRNA) platforms to further enhance intratumoral cytokine expression while maintaining a favorable safety profile. Unlike standard mRNA, srRNA is derived from alphavirus replicons and encodes both the gene of interest and viral nonstructural proteins that drive intracellular RNA amplification (123). This self-amplification enables sustained, high-level cytokine expression at substantially lower input doses, thereby addressing key limitations posed by mRNA instability and inefficient translation.

Li et al. demonstrated that lipid nanoparticles encapsulating srRNA encoding IL-12 induced potent antitumor immunity when administered intratumorally across multiple syngeneic tumor models (124). A single administration induced robust type I interferon signaling, dendritic cell activation, and immunogenic cancer cell death, resulting in strong infiltration of CD8^+^ T cells and NK cells into the TME. Importantly, this local immune activation propagated systemically, resulting in regression of untreated distant tumors and the establishment of long-term immune memory, while circulating IL-12 and IFN-γ levels remained below toxicity thresholds (124). These findings established srRNA-LNPs as an effective strategy that couples localized cytokine expression with durable systemic antitumor immunity.

A 2024 study extended this concept by demonstrating that systemically administered srRNA-LNPs encoding IL-12 can safely achieve therapeutically relevant intratumoral cytokine expression (84). Intravenous delivery of the srRNA formulation led to preferential accumulation and expression within tumors, where it remodeled the tumor microenvironment by reducing suppressive myeloid populations and enhancing infiltration and activation of cytotoxic lymphocytes. Notably, treatment induced significant tumor regression and prolonged survival in multiple solid tumor models and was well tolerated in both mice and non-human primates. When combined with PD-1 blockade, srRNA-IL-12 therapy showed synergistic efficacy, further supporting its potential as a modular platform for combination immunotherapy (84).

Although lipid nanoparticle-encapsulated IL-12 mRNA offers considerable promise for enhancing localized antitumor immune responses in cancer immunotherapy, its clinical adoption faces substantial hurdles, primarily the mRNA’s inherent instability to ribonucleolytic degradation and inefficient endosomal escape, which impair protein translation and efficacy (125–127). This technique is affected by immunogenicity from lipid nanoparticles and IL-12, which risks cytokine release syndrome and inflammation despite targeted delivery; by dosing complexities requiring personalized regimens to address tumor microenvironment and immune variations; and by poor targeting, leading to off-target effects and systemic uptake (128–131). Scalable manufacturing challenges, including batch variability and regulatory obstacles that raise costs and limit access, further emphasize the need for advances in nanoparticle design, biomarker-guided strategies, and clinical trials to overcome these barriers and realize IL-12 mRNA’s potential in oncology (125, 132).

#### 4.4.2 Polymer-based nanoparticles

Polymer-based nanoparticles represent a versatile class of non-viral IL-12 delivery vehicles, offering broad flexibility in formulation design, controllable release kinetics, and the capacity to carry either IL-12-encoding nucleic acids or recombinant protein. Two principal architectures have been investigated: polymersomes, stable, membrane-enclosed vesicles formed from amphiphilic block copolymers, and polyamidoamine (PAMAM) dendrimers, which provide a branched cationic scaffold for nucleic acid complexation and cellular delivery. Compared with liposomal platforms, polymersomes exhibit superior mechanical stability, slower cargo release, and greater resistance to degradation in the tumor microenvironment, properties that favor sustained intratumoral IL-12 exposure (133, 134). PAMAM dendrimers, conversely, enable efficient nucleic acid complexation and endosomal escape through their high cationic charge density. However, this property is associated with membrane disruption, hematological toxicity, and non-specific biodistribution (135, 136), a particular concern given that hepatotoxicity was the dose-limiting toxicity of systemic recombinant IL-12 in early clinical trials. Mitigation strategies include partial PEGylation to shield excess charge, lower-generation dendrimer formulations, and the use of biodegradable polymer backbones that degrade to non-toxic metabolites following cargo release (137).

Among the polymer scaffolds investigated, poly(beta-amino esters) (PBAEs) have attracted particular interest owing to their intrinsic biodegradability and established safety relative to non-degradable cationic polymers. Engineered PBAE nanoparticles co-loaded with the co-stimulatory ligand 4-1BBL and soluble IL-12 created a dual-signal depot that simultaneously drives T cell co-stimulation and Th1 polarization within the TME; combined with systemic PD-1 blockade, this platform induced tumor regression, complete clearance, and resistance to distant rechallenge (138). Beyond single-cytokine approaches, polymer platforms have been developed to co-deliver IL-12 alongside chemotherapeutic agents. A polymeric micelle cluster (HC/pIL-12/polyMET) co-delivering cisplatin and an IL-12-encoding plasmid exploited the synergy between chemotherapy-induced immunogenic cell death and IL-12-driven immune priming, significantly inhibiting tumor growth and prolonging survival in Lewis lung carcinoma models through increased IFN-γ secretion and M2-to-M1 macrophage polarization (139). An analogous system (HA/pIL-12/DOX-PMet) co-delivering doxorubicin and IL-12 in a hyaluronic acid-coated nanosystem achieved CD44-targeted tumor accumulation, relevant to PDAC, where CD44 is overexpressed, enhancing NK cell and CTL activity, suppressing Tregs, and producing high antitumor and antimetastatic efficacy in breast cancer models (140).

Two further studies illustrate how polymer engineering can address pharmacological limitations of IL-12. In a 2021 study, a surface-modified generation-5 PAMAM dendrimer was generated, combining alkyl chains for membrane destabilization with a low-molecular-weight protamine peptide for nuclear localization to transfect an IL-12-encoding plasmid into mesenchymal stem cells (MSCs) (136). The modified dendrimer achieved superior transfection efficiency and reduced cytotoxicity compared with unmodified PAMAM variants, and engineered MSCs retained preferential migration toward cancer cells, positioning them as tumor-homing cellular vehicles for IL-12 gene delivery. Addressing the distinct problem of systemic toxicity, an engineered pH-sensitive nanocytokine (Nano-IL-12) was created by encapsulating native IL-12 within a CDM-modified PEG-poly(L-lysine) polymer shell, forming pH-sensitive amide bonds that occlude receptor-binding epitopes at physiological pH and cleave under mild intratumoral acidosis (141). Administered intravenously in cold melanoma and triple-negative breast cancer models, Nano-IL-12 induced potent localized Th1 inflammation at approximately ten times lower doses than free IL-12, while suppressing systemic cytokine elevation and the secondary IL-10 counter-response responsible for clinical tachyphylaxis. Combined with anti-PD-1 and anti-CTLA-4 checkpoint inhibitors, Nano-IL-12 achieved complete eradication of ICI-resistant primary tumours and lung metastases, a directly analogous cold-tumor setting to PDAC (141).

Recently, a strategy integrating both targeted delivery and sustained local release demonstrated that covalent anchoring of IL-12 to the phospholipid headgroups of a poly-L-glutamate/poly-L-arginine layer-by-layer (LbL) liposomal nanoparticle via a maleimide–cysteine linkage enables a “target-and-release” mechanism critical for therapeutic efficacy in disseminated ovarian cancer (142). The PLE outermost layer mediates rapid and selective binding to ovarian cancer cell surfaces, while serum proteins in the interstitial tumor fluid gradually extract IL-12-conjugated lipids from the liposomal membrane over 24–48 hours, disseminating active cytokine throughout the tumor bed. In the ICI-resistant HM-1 ovarian cancer model, Mal LbL NPs produced a 50-fold increase in intratumoral CD8+ T cell infiltration and M2 macrophage repolarization. Combined with dual checkpoint blockade, the platform achieved 100% cure rates with durable immunological memory, attributable to IL-12 resolving the upstream immune exclusion barrier that renders checkpoint inhibitors ineffective in cold tumors, a mechanism of direct relevance to PDAC (142).

Despite these advances, polymer-based IL-12 carriers face shared translational challenges such as non-stable manufacturing, comprehensive biodegradation and immunogenicity assessment, and identification of biomarkers predicting TME responsiveness (143). In the PDAC context specifically, the dense desmoplastic stroma represents an additional physical barrier to nanoparticle diffusion; surface functionalization targeting stromal components such as hyaluronan or collagen, or co-delivery of stromal- depleting agents, represent active strategies to address this limitation (144).

#### 4.4.3 Exosomes

Exosomes are endogenously produced extracellular vesicles, and their biological origin provides them with a fundamentally distinct set of properties relative to synthetic nanocarriers, which is a natural phospholipid membrane bilayer that confers innate biocompatibility and reduced immunogenicity, surface expression of endogenous membrane proteins that facilitate cell-to-cell communication and uptake, and an intrinsic capacity for tissue homing that can be further engineered through surface modification (145). These characteristics make exosomes a biologically motivated and mechanistically distinctive platform for localized IL-12 delivery, and clearly distinguish them from lipid-based synthetic nanoparticles despite the shared membrane-vesicle architecture.

The principal exosome-based IL-12 strategy under investigation involves surface display of IL-12 on the exosome membrane (exoIL-12), rather than its encapsulation within the vesicle lumen. Surface anchoring preserves IL-12 receptor-binding geometry and bioactivity while enabling the exosome membrane to serve as a pharmacokinetic buffer, slowing cytokine diffusion away from the injection site and substantially extending intratumoral retention relative to freely injected recombinant IL-12 (146). In murine tumor models, intratumoral administration of exoIL-12 produced markedly higher local IL-12 concentrations, greater CD8+ T cell activation, and superior tumor regression compared with equivalent doses of recombinant IL-12, without detectable elevation of systemic IL-12 or IFN-γ to levels associated with cytokine toxicity. Importantly, exoIL-12-treated animals developed durable immunological memory, with protection against tumor rechallenge observed in a subset of models, an outcome consistent with the induction of a systemic antitumor response seeded by the localized IL-12 signal (146).

A notable clinical translation of the exosome IL-12 concept is the investigational agent CDK-003 (exoIL-12), an exosome-surface-displayed IL-12 construct that obtained positive results in a Phase 1/​2a study in solid tumors (NCT05156229). The development of CDK-003 reflects recognition that the favourable preclinical pharmacokinetics of exoIL-12, high intratumoral retention, limited systemic spillover, preserved IL-12 bioactivity, constitute a meaningful clinical advantage over both systemic recombinant IL-12 and earlier localized formulations, and that the exosome platform is sufficiently manufacturable to support clinical-grade production (146).

Several challenges specific to exosome-based platforms must nonetheless be acknowledged. The manufacture of clinical-grade exosomes at scale remains technically demanding. The isolation of homogeneous exosome populations by ultracentrifugation or size-exclusion chromatography is inherently low-yield, and batch-to-batch variability in vesicle size distribution, surface protein composition, and IL-12 loading density poses significant quality control challenges for compliance (146). The absence of finalized regulatory guidance specific to extracellular vesicle therapeutics, which occupy a novel biological entity classification distinct from both small-molecule drugs and conventional biologics, and the testing for immunogenicity of exosomes derived from allogeneic sources introduces additional development uncertainty, particularly regarding the potency assays and release criteria that will be required for regulatory submission (147).

### 4.5 Immunocytokines

Immunocytokines are generated by genetically fusing IL-12 to tumor-targeting antibodies or antibody fragments, thereby concentrating cytokine activity within the TME and enhancing antitumor immunity while reducing off-target exposure (148, 149). Their efficacy depends on the selection of appropriate tumor-targeting moieties, and multiple targeting strategies have been explored (as described below), each reflecting distinct features of tumor biology and antigen expression patterns (149–151). In parallel, the molecular architecture of IL-12 immunocytokines strongly influences their pharmacokinetics, intratumoral penetration, and immunostimulatory capacity (151). Although clinical data for IL-12 immunocytokines in PDAC therapy remain limited, several preclinical studies demonstrate the relevance of this strategy in this disease context. Tumor-targeted IL-2 immunocytokines, such as L19-IL2, substantially enhanced immune infiltration and cytotoxic function in orthotopic PDAC models, resulting in significant tumor growth inhibition, even in chemotherapy-resistant tumors (152). Earlier work with ED-B–targeted IL-2 immunocytokines confirmed suppression of orthotopic pancreatic tumor growth and metastasis, accompanied by macrophage and NK cell recruitment (153). In addition, bispecific constructs like PD1-IL2v, which direct IL-2 activity toward PD-1–expressing T cells, synergized with radiation therapy to markedly expand functional CD8^+^ T cells and improve local and systemic antitumor responses in PDAC preclinical models (154). These studies indicate that immunocytokine strategies are biologically active in PDAC and justify further translational development.

#### 4.5.1 Tumor neovasculature–targeted

Targeting tumor neovasculature exploits the accessibility and biological distinctiveness of tumor-associated vessels. One of the most extensively studied targets for IL-12 immunocytokines is the extra-domain B (ED-B) of fibronectin, a splice variant selectively expressed in tumor neovasculature but largely absent from normal adult tissues (149, 155). The F8 antibody, which recognizes ED-B fibronectin, has been used to generate IL-12 immunocytokines that selectively target tumor blood vessels (149, 155). Antibody-mediated delivery of IL12-F8-F8 to the tumor neovasculature induced robust antitumor responses across multiple murine cancer models, particularly when combined with paclitaxel (51). Significantly, unlike IL-2- or TNF-based immunocytokines, vascular-targeted IL12-F8-F8 did not exacerbate the inflammatory response. Mechanistically, the antitumor effects of neovasculature-targeted IL-12 were shown to depend on CD4^+^ T cells and were associated with pronounced interferon-γ (IFN-γ) production, highlighting the role of IL-12 in orchestrating coordinated cellular immune responses within the tumor milieu (51). In a previous study using subcutaneous xenograft models, huBC1-muIL12 treatment markedly slowed tumor growth compared with equivalent doses of free murine IL-12 or antibody plus IL-12 combinations, indicating that the antibody–cytokine fusion enhanced antitumor efficacy, likely by focally delivering IL-12 at ED-B–positive tumor sites. Although these models lack T and B cells, the potent antitumor effects were linked to activation of innate effector mechanisms such as NK cells and macrophages by the locally concentrated IL-12, and the improved performance of the fusion protein over free cytokine suggests that ED-B targeting increases delivery and functional impact within the TME (156). These studies validate ED-B fibronectin as a promising anchor for immunocytokine-mediated cytokine delivery.

#### 4.5.2 Necrosis-targeted

An alternative targeting strategy targets necrotic regions commonly found in solid tumors. The NHS-IL12 immunocytokine employs the NHS76 antibody, which binds exposed DNA–histone complexes in necrotic tumor tissue (148, 157). This approach leverages the pathological features of advanced tumors to achieve selective cytokine accumulation. The first-in-human Phase I trial of NHS-IL12 (NCT01417546) evaluated the safety and preliminary efficacy of NHS-IL12 in patients with metastatic solid tumors refractory to standard therapies (158).

A notable feature of necrosis-targeted IL-12 is its enhanced accumulation in tumors following radiotherapy. A study with murine xenograft models showed that local irradiation increased NHS-IL12 binding, suggesting that radiation-induced necrosis can augment intratumoral cytokine delivery (157). Mechanistically, antitumor activity was shown to be CD8^+^ T cell–dependent, both as monotherapy and in combination regimens, and was associated with tumor senescence and differentiation in humanized mouse models (157). A separate preclinical study further characterized the temporal immune remodeling induced by NHS-IL12 in bladder cancer models, revealing a shift from an immunosuppressive, myeloid-dominated TME toward enhanced T-cell activity (148).

Given the immunosuppressive nature of many solid tumors, IL-12 immunocytokines are increasingly evaluated in combination with immune checkpoint inhibitors. Preclinical studies combining NHS-IL12 with PD-1 or PD-L1 blockade demonstrated synergistic antitumor effects, accompanied by increased IFN-γ production and T-cell activation (76). These findings have translated into early clinical evaluation, with phase Ib trials investigating M9241 (NHS-IL12) in combination with avelumab in patients with advanced solid tumors (NCT02994953) (53).

#### 4.5.3 Stromal and tumor antigen–targeted

Beyond vascular and necrotic targeting, IL-12 immunocytokines have been developed to target stromal and tumor-associated antigens. Fibroblast activation protein (FAP), highly expressed on cancer-associated fibroblasts and minimally expressed in normal tissues, represents an attractive stromal target (159). In murine renal and melanoma models, administration of mIL12-7NP2 (FAP targeted) demonstrated potent antitumor activity and enhanced the accumulation of NK cells and CD8+ T cells in the tumor mass. Moreover, human IL12-7NP2 tested in non-human primates was well tolerated at the tested dose levels, with no major perturbations in overall lymphocyte or B-cell percentages observed over the course of treatment and no overt clinical signs of toxicity, supporting an acceptable safety profile for further clinical development (159).

Tumor cell–specific targets have also been explored. Mesothelin-targeted IL-12 immunocytokines, such as IL12-SS1(Fv), inhibited mesothelioma growth in preclinical models and highlighted the feasibility of antigen-restricted IL-12 delivery in malignancies with high mesothelin expression (149). Similarly, HER2-targeted IL-12 immunocytokines incorporating heterodimeric Fc designs have demonstrated enhanced tumor penetration and reduced Fcγ receptor–mediated off-target effects, resulting in improved antitumor efficacy (151).

Despite encouraging preclinical and early clinical findings, several limitations continue to constrain the development and clinical translation of IL-12 immunocytokines. A primary challenge arises from the heterodimeric structure of IL-12, composed of disulfide-linked p35 and p40 subunits, which complicates fusion protein design, manufacturing stability, and product homogeneity compared with monomeric cytokines (155). Different fusion formats exhibit distinct biodistribution and biological activity profiles, and vector design, as well as gene dosage effects, can significantly influence production yield and quality (155, 160). In addition, achieving efficient intratumoral penetration while maintaining favorable pharmacokinetics remains a major challenge. Recent engineering solutions, such as heterodimeric Fc designs that minimize Fcγ receptor binding, have improved tumor penetration and reduced off-target clearance, but further optimization is required to balance tumor accessibility with systemic exposure (151). Beyond structural considerations, the immunosuppressive tumor microenvironment represents a dominant biological barrier. Tumor-intrinsic escape mechanisms and suppressive immune networks can attenuate or override IL-12–driven antitumor immunity, even when targeted delivery successfully induces tumor-specific immune responses (80). These factors likely contribute to the discrepancy between robust preclinical efficacy and more modest clinical outcomes observed to date (161). Finally, the optimal positioning of IL-12 relative to other cytokine payloads and combination partners remains incompletely defined. Determining which tumor indications, delivery platforms, and combinatorial regimens are best suited for IL-12–based immunocytokines will be essential to improve therapeutic index and maximize clinical benefit (162).

### 4.6 Local electroporation-mediated transfection

Intratumoral delivery of IL-12-encoding plasmid DNA followed by electroporation (EP) represents one of the most clinically advanced localized IL-12 strategies and one of the few approaches with substantial human efficacy and safety data across multiple tumor types. The mechanistic rationale for this platform rests on electroporation capacity to transiently permeabilize cell membranes, enabling direct plasmid uptake and robust transgene expression within the treated lesion while confining IL-12 production, and its downstream immunological consequences, to the tumor environment (50, 163). This titratable, spatially restricted mode of cytokine delivery enables the IL-12–IFN-γ–CXCR3 axis to operate locally, driving intratumoral Th1 polarization, CD8+ T cell infiltration, and antigen presentation machinery upregulation without the supraphysiologic serum cytokine concentrations that produced toxicity in early systemic recombinant IL-12 trials (164, 165).

Foundational preclinical work established that *in vivo* EP delivery of an IL-12 plasmid could drive tumor regression and systemic antitumor immunity in murine models (166, 167). This mechanistic principle was directly translated into a Phase I trial in patients with metastatic melanoma, in which intratumoral IL-12 plasmid EP established the safety and feasibility of the approach in humans: treatment-related adverse events were predominantly transient local pain, serum IL-12 levels remained limited, and treated lesions demonstrated dose-proportional increases in intratumoral IL-12 protein with accompanying tumor necrosis and lymphocytic infiltration (50). A subset of patients achieved local disease stabilization or partial responses, providing the first clinical proof of concept for EP-mediated IL-12 gene transfer as a tolerable and biologically active platform.

Subsequent investigation supported this evidence considerably when intratumoral delivery of tavokinogene telseplasmid (tavo) via EP in patients with advanced melanoma produced objective responses alongside systemic immunological remodeling, including upregulation of immune transcripts and increased CD8+ tumor-infiltrating lymphocytes in both injected and non-injected lesions, strongly supporting the systemic immunological reach of locally administered IL-12 EP (163, 168). The observation of immune activation in untreated distant suggests that local IL-12 transfection initiates a systemic effector T cell response analogous to the abscopal effect described with radiotherapy, and that this systemic component can be further amplified through combination with checkpoint blockade. Consistent with this, a neoadjuvant study combining intratumoral tavo-EP with Pembrolizumab in advanced melanoma observed high pathological complete response, providing early clinical evidence of synergy between localized IL-12 transfection and systemic PD-1 inhibition (169).

Beyond melanoma, intratumoral IL-12 EP has demonstrated clinical activity in Merkel cell carcinoma (MCC), a virally associated and immunologically relevant skin cancer. In a pilot trial in MCC, tavo-EP produced sustained intratumoral IL-12 expression, augmented CD8+ T cell infiltration, systemic immune activation, and clinical responses, including regression of uninjected lesions, an abscopal pattern paralleling the melanoma experience, and reinforcing the *in situ* vaccination mechanism (170). Across both tumor types, the toxicity profile was consistently characterized by local injection-site reactions and transient inflammation, with systemic IL-12-mediated toxicities absent in the majority of evaluable patients.

Despite its clinical promise, intratumoral IL-12 electroporation remains constrained by important limitations. The most commonly reported adverse event is transient procedure-related pain, which may accumulate with repeated administrations (50, 163, 168, 170). Moreover, this approach is restricted to superficial, accessible lesions, limiting applicability in patients with visceral or anatomically inaccessible tumors. Although local tumor regression is frequently observed, systemic antitumor immunity and abscopal responses remain variable and often insufficient for durable control of distant disease (168, 171, 172). Gene transfer efficiency, while improved compared to naked DNA injection, remains heterogeneous across tumor types and patients (50, 170, 173). Finally, systemic leakage of IL-12 can still occur despite localized delivery, with potential toxicity at higher doses, underscoring the challenge of fully confining IL-12 activity to the tumor site (174). Collectively, these limitations highlight the need for further research into controllable, broadly applicable IL-12 electroporation delivery platforms.

### 4.7 Combined IL-12 and CAR-T cell

CAR-T cell therapy has demonstrated transformative efficacy in hematological malignancies. However, its translation to solid tumors, including PDAC, has been fundamentally constrained by the immunosuppressive TME, which impairs CAR-T cell trafficking, persistence, and effector function within the tumor parenchyma. The principal challenge, however, remains delivering sufficient IL-12 activity to the TME without incurring the systemic toxicities that halted early clinical programmes. This challenge has driven the development of CAR-T constructs engineered to produce IL-12 in a spatially restricted, tumor-confined manner.

The most advanced approach in this category involves membrane-bound IL-12 (mbIL-12)-armored CAR-T cells, in which IL-12 is expressed on the CAR-T cell surface rather than secreted freely into the circulation. mbIL-12-engineered CAR-T cells administered locoregionally in ovarian cancer xenograft models produced antigen-dependent proliferation, superior intratumoral effector activity, and a favourable safety profile compared with soluble IL-12 strategies, with systemic IL-12 exposure remaining below toxic thresholds (175). The membrane-anchored configuration restricts IL-12 signaling to the immediate tumor microenvironment, enabling robust local IFN-γ production and Th1 polarization without indiscriminately activating circulating immune cells. A combination of mbIL-12 CAR-T cells with PD-L1 blockade demonstrated additive to synergistic antitumor activity in preclinical models, consistent with the mechanistic complementarity described for other localized IL-12 platforms. IL-12 resolves T cell exclusion, while checkpoint inhibition rescues exhausted effectors (175).

A distinct and innovative approach employs collagen-binding domain IL-12 (CBD-IL-12) fusion proteins in combination with antigen-targeted CAR-T cells. In advanced prostate cancer models, STEAP1-targeted CAR-T cells combined with CBD-IL-12, a fusion protein that tethers IL-12 to collagen within the desmoplastic tumor stroma, produced TME remodeling, reduced STEAP1 antigen escape through epitope spreading, and synergized with checkpoint inhibition to eradicate large tumors in mouse models (78, 176). By engaging host immune cells beyond the CAR-T target antigen, CBD-IL-12 also broadens the antitumor immune response, potentially mitigating antigen escape, a major mechanism of CAR-T failure in solid tumors (78, 176).

An alternative engineering strategy employs CRISPR activation (CRISPRa) to upregulate endogenous IL-12 subunit expression within HER2-targeted CAR-T cells in an antigen-dependent manner. Using CRISPRa, the inducible, autocrine IL-12 circuit enhanced IFN-γ production, CAR-T cytotoxicity, and *in vivo* tumor control. At the same time, systemic IL-12 remained undetectable, underscoring the precision achievable when IL-12 production is directly coupled to CAR-T antigen recognition and confined to tumor-engagement sites (177). Combination with PD-L1 blockade further enhanced antitumor activity in this system, again reinforcing the synergistic logic of pairing upstream T cell recruitment and activation with downstream checkpoint relief (177).

Despite its efficacy in hematologic malignancies, CAR T cell therapy remains constrained by substantial clinical, manufacturing, logistical, economic, and regulatory limitations. Severe toxicities, including CRS and immune effector cell–associated neurotoxicity syndrome, require specialized inpatient management and restrict broader accessibility (178, 179). Relapse driven by antigen escape, limited CAR T cell persistence, and T cell exhaustion further undermine the long-term durability of response, particularly in CD19- and BCMA-targeted therapies (180–182). Moreover, efficacy in solid tumors remains limited due to antigen heterogeneity and the immunosuppressive tumor microenvironment (181). The autologous manufacturing process is complex, time-consuming, and vulnerable to failure, creating delays that may necessitate bridging therapy and expose clinically unstable patients to disease progression (183). Finally, the exceptionally high cost of therapy remains a profound barrier to equitable access (178). Collectively, while CAR T cell therapy represents a major milestone in cellular immunotherapy, its complexity, toxicity profile, and systemic infrastructure demands highlight the need for more scalable, accessible, and controllable next-generation immune-engineering strategies.

## 5 Discussion

Interleukin-12 has long been recognized as one of the most potent cytokines for generating antitumor immunity, yet the persistent challenge of systemic toxicity has shaped its clinical trajectory. Across solid tumors, high circulating IL-12 levels induce severe immune-related adverse events that prevent safe dose escalation and limit biological activity. This historical limitation explains the gap between IL-12’s strong mechanistic rationale and its limited clinical success. Our literature review indicates that the central issue is not IL-12 itself, but rather the inability of early delivery methods to achieve spatially restricted, temporal, and dose-controlled cytokine exposure within the tumor microenvironment.

Recent technological advances have reinstated interest in IL-12 as a therapy by enabling delivery strategies that fundamentally expand its therapeutic window. Encapsulation systems, virotherapy vectors, and lipid vectors collectively demonstrate a shift away from systemic administration of recombinant IL-12 toward platforms engineered to contain IL-12 activity within tumor or stromal compartments. These modalities show promise in reducing off-target exposure, enabling sustained but controlled cytokine release, and enhancing functional interaction with effector immune cells. Notably, these delivery systems not only address toxicity but also overcome biological barriers that are particularly relevant for PDAC, including dense desmoplasia, poor vascularity, and profound immune exclusion. However, while pre-clinical studies consistently show improved safety profiles and enhanced T and NK cell activation, early-phase clinical data remain limited.

A recurring theme across delivery platforms is that IL-12 is unlikely to be effective as a monotherapy. Its most promising applications appear to be in combination settings that exploit IL-12’s capacity to remodel suppressive microenvironments and potentiate other immunotherapies. For PDAC, therapeutic combinations could include immune checkpoint inhibitors, stromal-modulating agents, targeted therapies that modulate immunometabolism, or CAR-T and NK cell approaches that benefit from IL-12-driven Th1 polarization and enhanced cytotoxicity. The integration of IL-12 with currently ineffective immunotherapies for PDAC may enhance their activity by rendering tumors more immunologically accessible. The challenge lies in sequencing and timing to determine whether IL-12 should prime the immune system before other interventions or amplify responses already initiated. Early-phase clinical trials exploring these combinations must incorporate sophisticated immune monitoring to dissect mechanisms and identify optimal scheduling.

Nevertheless, several knowledge gaps must be addressed to guide next-generation IL-12 strategies. These include identifying biomarkers predictive of IL-12 responsiveness, understanding the kinetics of IL-12–induced immune activation across different tumor architectures, and determining optimal dosing frequencies to sustain antitumor immunity without triggering cytokine-related toxicities. The concept of personalized IL-12 therapy, based on individual tumor immune phenotypes characterized through comprehensive immune profiling, remains aspirational but increasingly feasible as technologies mature and costs decline. Additionally, the practical challenges beyond scientific innovation related to the manufacturing complexity of these platforms raise questions about scalability and cost that will ultimately determine accessibility. Regulatory pathways for combination therapies involving multiple novel components remain uncertain, potentially delaying clinical development. The long-term safety profile of sustained IL-12 exposure, even when localized, requires vigilant monitoring in extended follow-up studies, as autoimmune phenomena or chronic inflammatory states could emerge over time. Additionally, PDAC’s propensity for early metastatic dissemination means that even optimal local IL-12 delivery must generate systemic immunity sufficient to control distant disease.

In summary, IL-12 is re-emerging as a viable immunotherapeutic agent not because its biology has changed, but because delivery technologies have evolved to meet the demands of its potent activity. Continued innovation in localized and controlled IL-12 delivery, together with thoughtful combinatorial approaches, holds significant promise for countering the profound immunosuppression characteristic of PDAC and other refractory solid tumors. The field now possesses tools capable of unlocking IL-12’s long-recognized therapeutic power, which will require coordinated efforts in mechanistic studies, biomarker development, and clinical translation.
